# Resistance, virulence and genetic diversity of *Salmonella* Typhimurium in South Africa (1999–2021)

**DOI:** 10.4102/ojvr.v92i1.2217

**Published:** 2025-10-06

**Authors:** Nkagiseng Moatshe, Emmanuel Seakamela, Khanyisile R. Mbatha, Linda A. Bester, Nombasa Ntushelo, Itumeleng Matle

**Affiliations:** 1 School of Interdisciplinary Research and Graduate Studies, College of Graduate Studies, University of South Africa, Pretoria, South Africa; 2 Department of Biotechnology, Onderstepoort Veterinary Research, Agricultural Research Council, Pretoria, South Africa; 3 Bacteriology Division, Onderstepoort Veterinary Research, Agricultural Research Council, Pretoria, South Africa; 4 Biomedical Resource Unit School of Laboratory Medicine and Medical Sciences, University of KwaZulu-Natal, Durban, South Africa; 5 Department of Biometry, Infruitec, Agricultural Research Council, Cape Town, South Africa; 6 Department of Agriculture and Animal Health, College of Agriculture and Environmental Sciences, University of South Africa, Florida, South Africa

**Keywords:** *Salmonella*/salmonellosis, animal, environment, diarrheic FBD, zoonosis, antibiotic resistance, ERIC PCR

## Abstract

*Salmonella* Typhimurium is a major cause of human and animal salmonellosis, impacting global socioeconomic factors. This study examined antibiotic resistance patterns, virulence genes and plasmids in *S.* Typhimurium isolates from nine South African provinces (1999–2021). Among 180 randomly selected isolates, 129 were confirmed as *S.* Typhimurium from animal (*n* = 94), food (*n* = 16), environment (*n* = 7) and feed (*n* = 12) sources using polymerase chain reaction (PCR). Phenotypic resistance was assessed against 13 antibiotics, revealing high resistance levels: 86.8% to ciprofloxacin, 69.0% to ceftriaxone and 65.1% to piperacillin. Multidrug resistance (up to 11 antibiotics) was observed. Genetic analysis identified resistance genes, including *bla*_PSE_ (32.6%), *bla*_CMY-2_ (21.7%), *tetA* (24.0%), *tetB* (22.5%), *qnrB* (21.7%) and *qnrA* (20.2%). Class 1 integrons were found in 47.4% of isolates. Virulence genes were prevalent, including *sopB* (95.3%), *sspH1* (82.9%), *sifA* (82.9%), *pefA* (79.8%), *spvC* (79.1%), *sspH2* (77.5%), *srgA* (71.3%) and *invA* (100%). Plasmid analysis detected 2 kb, 8 kb and 90 kb plasmids, with the 90 kb plasmid being most common (71.3%). Enterobacterial Repetitive Intergenic Consensus (ERIC) PCR identified 44 clusters (A–RR), including 6 major clusters.

**Contribution:**

These findings highlight the urgent need for enhanced surveillance and intervention strategies to curb antibiotic resistance and virulence in *S.* Typhimurium populations in South Africa, stressing the importance of monitoring and control measures to address this public health threat.

## Introduction

*Salmonella* Typhimurium is a Gram-negative, facultative anaerobic bacterium belonging to the family *Enterobacteriaceae*. It is a leading serovar implicated in salmonellosis, a zoonotic disease of considerable public health and economic importance globally (Galán-Relaño et al. 2023). Alongside *S. Enteritidis, S*. Typhimurium is one of the most frequently reported serotypes in South Africa, contributing significantly to the global burden of foodborne illnesses, with estimated 93.8 million human cases annually (Majowicz et al. 2010; Ramatla et al. 2022).

In humans, *S*. Typhimurium infection typically results from consuming contaminated food products derived from animals, particularly poultry, eggs and dairy. The bacterium can also be transmitted via the faecal–oral route (Mkangara 2023). *Salmonella* Typhimurium infection in humans often manifests as self-limiting gastroenteritis characterised by diarrhoea, abdominal pain, vomiting and fever, but in immunocompromised individuals or those with co-morbidities, it can lead to severe invasive disease, such as bacteraemia or systemic septicaemia (Wright et al. 2005). The invasive non-typhoidal *S.* Typhimurium strains (iNTS), particularly those prevalent in sub-Saharan Africa, pose heightened risks because of their ability to cause life-threatening systemic disease in vulnerable populations, including infants and human immunodeficiency virus (HIV)-positive individuals (Hajra, Nair & Chakravortty 2023; Okoro et al. 2012).

In animals, *S*. Typhimurium infections present diverse clinical manifestations ranging from asymptomatic carriage to severe systemic disease, such as septicaemia or enteritis. These infections pose challenges to animal health, agricultural productivity and food safety, with livestock serving as both reservoirs and amplifiers of the pathogen (Hoelzer, Moreno Switt & Wiedmann 2011). The adaptability of *S*. Typhimurium to environmental stressors and host immune responses is underpinned by complex regulatory systems governing gene expression, enabling survival and replication across diverse ecological niches (Ilyas, Tsai & Coombes 2017).

The pathogenicity of *S*. Typhimurium is mediated by numerous virulence factors encoded on the chromosomal pathogenicity islands (SPIs) and plasmids. SPI-1 facilitates host cell invasion through a type III secretion system (T3SS), delivering effector proteins that induce cytoskeletal rearrangements and membrane ruffling in epithelial cells (Lou et al. 2019). SPI-2, another critical virulence determinant, enables intracellular survival and replication within macrophages, protecting the bacterium from immune-mediated clearance (Ramatla et al. 2020). In addition, the 90-kb pSLT virulence plasmid encodes genes such as *spvRABCD*, which enhance systemic infection, and *rck* and *pef*, which confer resistance to complement-mediated killing and promote adhesion, respectively (Rotger & Casadesús 1999; Silva, Puente & Calva 2017).

The emergence and dissemination of multidrug resistant (MDR) *S*. Typhimurium strains have exacerbated the global public health challenge, complicating treatment options. Resistance is often associated with mobile genetic elements such as plasmids, transposons and integrons. Class 1 integrons, commonly identified in *S*. Typhimurium, harbour gene cassettes encoding resistance to multiple antibiotics, including sulphonamides (*sul1, sul2*), chloramphenicol (*cat1, cat2*) and tetracyclines (*tetA, tetB*) (Gillings 2014; Wang et al. 2014). Resistance to critical drugs such as β-lactams (*bla*CTX-M, *bla*TEM-1, *bla*SHV) and fluoroquinolones (*qnrA, qnrB*) further complicates management strategies (Adesiji et al. 2014; Eguale et al. 2017). The global spread of MDR *S*. Typhimurium phage type DT104, known for its resistance to at least five antibiotics, underscores the urgency of addressing antimicrobial resistance (Wang et al. 2019; Threlfall 2002).

Despite the significant burden of *S*. Typhimurium infections, data on its prevalence, epidemiology and economic impact in sub-Saharan Africa remain fragmented. In South Africa, the lack of integrated surveillance systems limits comprehensive understanding and control of this pathogen in both human and animal populations (Mthembu, Zishiri & El Zowalaty 2019). Surveillance efforts rely heavily on laboratory reports from abattoirs, diseased animals and feed monitoring, which provide valuable insights but fail to capture the full scope of its impact (Kidanemariam et al. 2010; Khumalo & Mbanga 2014). The overuse and misuse of antibiotics in livestock exacerbate the dissemination of resistant strains, posing a dual threat to public health and food security by affecting livestock productivity and the safety of animal-derived products.

Therefore, the aim of this study was to investigate the antibiotic resistance patterns, virulence gene profiles and genetic diversity of *S*. Typhimurium isolated from the environment, animals and food products in South Africa over a 22-year period (1999–2021). By analysing isolates from diverse sources, the study sought to elucidate the prevalence of antimicrobial resistance, characterise virulence determinants and assess the genetic relatedness of strains, contributing to a deeper understanding of the epidemiology of *S.* Typhimurium and its implications for public health, food safety and antimicrobial stewardship in the region.

## Research methods and design

### Study design and isolates selection

The study was a retrospective cohort analysis based on laboratory-confirmed isolates of *S*. Typhimurium collected from diverse sources across South Africa between 1999 and 2021. These isolates were initially recovered from samples submitted for diagnostic purposes and preserved by freeze-drying, ensuring long-term viability. Samples were stored at the General Bacteriology Laboratory of the Onderstepoort Veterinary Research, South Africa. Based on the simple random sampling without replacement, a total of 180 isolates of *S*. Typhimurium from (1) various geographical locations in the country, (2) different sources of isolation (animal, animal products, feed and environmental samples) and (3) animal species (livestock, companion animals, wildlife) were included in this study. To prepare the isolates for further analysis, each freeze-dried sample was carefully revived. The isolates were inoculated into a brain heart infusion (BHI) broth and incubated at 37 °C for 18 h – 24 h to restore bacterial viability and ensure optimal growth conditions for downstream testing.

### Deoxyribonucleic acid extraction and polymerase chain reaction confirmation of *S*. Typhimurium

The genomic deoxyribonucleic acid (DNA) was extracted from culture using the boiling method as described by Karimnasab et al. (2013). Isolates were confirmed using a multiplex polymerase chain reaction (PCR) as described by Kim et al. (2006). In brief, a 25 µL-PCR mixture comprising 10.5 µL of Taq 2× Master Mix RED (Ampliquor, Denmark), 0.5 µL (5 pmol/µL) of each primer (Inqaba Biotechnical Industries (Pty) Ltd., South Africa) (Table 1), 4.5 µL UltraPure DNase/RNase-Free Distilled Water (Thermo Fisher Scientific, United States [US]) and 4 µL DNA template. The thermocycler (A 9700 Applied Biosystems, US) was set as follows: initial denaturation at 94 °C for 5 min, 40 cycles of denaturation at 94 °C for 30 s, annealing temperature 62 °C for 30 s, extension at 72 °C for 1 min and final extension at 72 °C for 3 min. The PCR amplicons were analysed by electrophoresis on a 1.5% agarose gel containing 4 µL ethidium bromide using ultraviolet (UV) light and photographed (Omega Fluor, Aplegen). *Salmonella* Typhimurium (ATCC 14028) strain and ribonuclease/deoxyribonuclease (RNase/DNase) free water were used as a positive and negative controls, respectively.

**TABLE 1:** Primers used for confirmation of *Salmonella* Typhimurium isolates.

Primer	Primer sequence (5’ to 3’)	Size (bp)
STM0716F	AACCGCTGCTTAATCCTGATGG	187
STM0716R	TGGCCCTGAGCCAGCTTTT	
STM1350F	TCAAAATTACCGGGCGCA	171
STM1350R	TTTTAAGACTACATACGCGCATGAA	
STM0839F	TCCAGTATGAAACAGGCAACGTGT	137
STM0839R	GCGACGCATTGTTCGATTGAT	
STM4525F	TGGCGGCAGAAGCGATG	114
STM4525R	CTTCATTCAGCAACTGACGCTGAG	
STM4538F	TGGTCACCGCGCGTGAT	93
STM4538R	CGAACGCCAGGTTCATTTGT	
STM2150F	CATAACCCGCCTCGACCTCAT	101
STM2150R	AGATGTCGTGAGAAGCGGTGG	

Source: Kim, S., Frye, J.G., Hu, J., Fedorka-Cray, P.J., Gautom, R. & Boyle, D.S., 2006, ‘Multiplex PCR-based method for identification of common clinical serotypes of *Salmonella enterica* subsp. *enterica*’, *Journal of Clinical Microbiology* 44, 3608–3615. https://doi.org/10.1128/JCM.00701-06

bp, base pair.

### Antimicrobial susceptibility testing

All isolates were subjected to antimicrobial susceptibility test using Kirby Bauer disk diffusion method and interpreted according to the European Committee on Antimicrobial Susceptibility Testing (EUCAST 2021). The isolates were tested against 13 antibiotics (Table 5) (Thermo Fisher Scientific, United Kingdom [UK]). These antibiotics were selected based on the consultation with veterinarians, the World Health Organization (WHO) ranking of antimicrobials in human medicine as well as the availability of EUCAST antibiotic breakpoints. In brief, the overnight pure cultureson nutrient agar were inoculated into sterile saline and diluted to the equivalent concentration of 0.5 McFarland standard. The bacterial suspension was inoculated aseptically onto Mueller–Hinton agar plates (Thermo Fisher Scientific, UK) and allowed to stand for 5 min. Antibiotic discs were then placed per inoculated plate followed by incubation at 37 °C for 24 h. The antibiotic susceptibility of each bacterial isolate was reported as sensitive or resistant according to the zone diameters described in the EUCAST (2021).

### Determination of antibiotic-resistant genes

Various multiplex PCR were performed for the screening of 18 resistance genes (Table 2). The 25 µL-PCR reaction mixture comprised 12.5 µL of Taq 2× Master Mix RED (Ampliquor, Denmark), 0.512 µL (5 pmol/µL) of each primer (Inqaba Biotechnical Industries (Pty) Ltd., South Africa), 4.5 µL (with the exception β-lactams 3.5 µL and tetracycline 5.5 µL) UltraPure DNase/RNase-Free Distilled Water (Thermo Fisher Scientific, US) and 5 µL DNA template. The thermocycler (Applied Biosystems, US) was set as follows: initial denaturation at 94 °C for 3 min, 30 cycles of denaturation at 94 °C for 30 s, annealing temperature variable (Table 2) for 30 s, extension at 72 °C for 1 min and final extension at 72 °C for 10 min. The PCR amplicons were analysed by electrophoresis on a 2.5% agarose gel containing 4 µL ethidium bromide using UV light and photographed (Omega Fluor, Aplegen).

**TABLE 2:** Primers annealing temperatures and expected base sizes.

Antibiotic class	Gene	Primer sequence (5’ to 3’)	Size (bp)	Melting temperature (^o^C)	Reference
Tetracycline	*tetA*	F:GGCGGTCTTCTTCATCATCATGC R:CGGCAGGCAGAGCAGTAGA	502	59	Pavelquesi et al. (2021)
	*tetB*	F:CGCCCAGTGCTGTTGTTGTC R:CGCGTTGAGAAGAAGCTGAGGTG	173		
Trimethoprim	*dfrl*	F:CGGTCGTAACACGTTCAAGT R:CTGGGGATTTTCAGGAAAGTA	220	55	Matayoshi et al. (2015); Khakrizi et al. (2022)
	*dfrXII*	F:AAATTCCGGGTGAGCAGAAG R:CCCGTTGACGGAATGGTTAG	429		
	*dfrXIII*	F:GCAGTCGCCCTAAAACAACG R:GATACGTGTGACAGCGTTGA	294		
Sulphonamides	*sul1*	F:CGGCGTGGGCTACCTGAACG R:GCCGATCGCGTGAAGTTCCCG	433	63	Pavelquesi et al. (2021)
	*sul2*	F:GCGCTCAAGGCAGATGGCATT R:GCGTTTGATACCGGCACCCGT	293		
	*sul3*	F:CAACGGAAGTGGGCGTTGTGGA R:GCTGCACCAATTCGCTGAACG	244		
Phenicol	*cat1*	F:CTTGTCGCCTTGCGTATAAT R:ATCCCAATGGCATCGTAAAG	508	53	Odoch et al. (2018)
	*Flo*	F:CTGAGGGTGTCGTCATCTAC R:GCTCCGACAATGCTGACTAT	673		
	*cmlA*	F:CGCCACGGTGTTGTTGTTAT R:GCGACCTGCGTAAATGTCAC	394		
β-lactams	*bla*_TEM_	F:TTAACTGGCGAACTACTTAC R: GTCTATTTCGTTCATCCATA	247	55	Karolina et al. (2022)
	*bla*_CMY-2_	F:GACAGCCTCTTTCTCCACA R:TGGACACGAAGGCTACGTA	1000		
	*bla*_SHV_	F:AGGATTGACTGCCTTTTTG R:ATTTGCTGATTTCGCTCG	393		
	*bla*_PSE_	F:TGCTTCGCAACTATGCTAC R:AGCCTGTGTTTGAGCTAGAT	438		
Quinolones	*qnrA*	F:TCAGCAAGAGGATTTCTCA R:GGCAGCACTATTACTCCCA	516	53	Takaichi et al. (2022)
	*qnrB*	F:GATCGTGAAAGCCAGAAAGG R:ACGATGCCTGGTAGTTGTCC	469		
	*qnrS*	F:ACGACATTCGTCAACTGCAA R:TAAATTGGCACCCTGTAGGC	417		

Source: Please see the full reference list of the article Moatshe, N., Seakamela, E. & Mbatha, K.R., 2025, ‘Resistance, virulence and genetic diversity of *Salmonella* Typhimurium in South Africa (1999–2021)’, *Onderstepoort Journal of Veterinary Research* 92(1), a2217. https://doi.org/10.4102/ojvr.v92i1.2217, for more information

*tetA, tetB*, tetracyclines; *cat1, cat2*, chloramphenicol; *sul1, sul2*, sulphonamides; *bla*CTX-M, *bla*TEM-1, *bla*SHV, β-lactams; *qnrA, qnrB*, fluoroquinolones; bp, base pair.

### Molecular detection of integrons

Isolates were screened for *int1, int2* and *int3* genes carried by class 1, 2 and 3 integrons, respectively using PCR. Briefly, 25 µL PCR reactions used comprised 12.5 µL of Taq 2× Master Mix RED (Ampliquor, Denmark), 2 µL (10 mM) of each primer (Inqaba Biotechnical Industries (Pty) Ltd., South Africa), 4.5 µL UltraPure DNase/RNase-Free Distilled Water (Thermo Fisher Scientific, US) and 4 µL DNA template. A 9700 PCR machine (Applied Biosystems, US) was used for amplification using PCR conditions (Table 3). The PCR products were electrophoresed in 1.5% agarose gel containing 4 µL ethidium bromide at 120 V for 60 min and photographed under UV light (Omega Fluor, Aplegen).

**TABLE 3:** Integron primers and polymerase chain reaction cycling conditions.

Target gene	Primer	Primer sequence (5’ to 3’)	PCR conditions	Number of cycles	Size (bp)	Reference
*int1*	Int1F Int1R	GCCTTGCTGTTCTTCTACGG GATGCCTGCTTGTTCTACGG	94 °C for 5 min 94 °C for 30 s, 60 °C for 30 s, 72 °C for 2 min 72 °C for 5 min	35	558	Ramatla et al. (2022)
*int2*	Int2F Int2R	CACGGATATGCGACAAAAAGG TGTAGCAAACGAGTGACGAAATG	94 °C for 5 min, 94 °C for 1 min, 60°C for 1 min, 72 °C for 2 min 72 °C for 10 min	32	740	
*int3*	Int3F Int3R	GCCTCCGGCAGCGACTTTCAG ACGGATCTGCCAAACCTGACT	94 °C for 10 min, 94 °C for 40s, 59°C for 50 s, 72°C for 55 s 72 °C for 10 min	35	650	Rowe-Magnus and Mazel (2002)

Source: Ramatla, T., Mileng, K., Ndou, R., Mphuti, N., Syakalima, M., Lekota, K.E. et al., 2022, ‘Molecular detection of integrons, colistin and β-lactamase resistant genes in *Salmonella enterica* serovars enteritidis and Typhimurium isolated from chickens and rats inhabiting poultry farms’, *Microorganisms* 10(2), 1–13. https://doi.org/10.3390/microorganisms10020313; Rowe-Magnus, D.A. & Mazel, D., 2002, ‘The role of intégrons in antibiotic resistance gene capture’, *International Journal of Medical Microbiology* 292(2), 115–125. https://doi.org/10.1078/1438-4221-00197

### Determination of virulence genes

A screening for 15 virulence genes was also performed on all *S.* Typhimurium isolates (Table 4). The 25 µL PCR reaction mixture comprised 12.5 µL of Taq 2× Master Mix RED (Ampliquor, Denmark), 0.5 µL (5 pmoL/µL) of each primer (Inqaba Biotechnical Industries (Pty) Ltd., South Africa), 4.5 µL (with exception *sopB, gipA* and *sspH1* 6.5 µL) UltraPure DNase/RNase-Free Distilled Water (Thermo Fisher Scientific, US) and 4 µL DNA template. Amplification was carried out as described by Ntivuguruzwa (2016). In brief, a thermocycler (9700 Applied Biosystems, US) was set as follows: initial denaturation at 94 °C for 3 min, 30 cycles of denaturation at 94 °C for 30 s, annealing temperature variable (Table 4) for 1 min, extension at 72 °C for 1 min and final extension at 72 °C for 5 min. PCR amplicons were analysed by electrophoresis on a 1.5% agarose gel containing 4 µL ethidium bromide using UV light and photographed (Omega Fluor, Aplegen).

**TABLE 4:** Virulence genes primer sequences, expected and annealing temperature.

Gene	Melting temperature (°C)	Size (bp)	Primer sequence (5’ to 3’)	References
*sopB*	64	220	F-GGACCGGCCAGCAACAAAACAAAGAAGAAG R- TAGTGATGCCCGTTATGCGTGAGTGTATT	Skyberg, Logue and Nolan (2006) Ntivuguruzwa (2016)
*gtgB*	57.5	436	F – TGCACGGGGAAAACTACTTC R – TGATGGGCTGAAACATCAAA	Chiu and Ou (1996)
*invA*		244	F – ACAGTGCTCGTTTACGACCTGAAT R – AGACGACTGGTACTGATCGATAAT	
*sspH1*	60	246	F – TGCAGAAAAAGGGGAATACG R – GCAGCCTGAAGGTCTGAAAC	Capuano et al. (2013)
*sopE*	57.5	362	F – CGAGTAAAGACCCCGCATAC R – GAGTCGGCATAGCACACTCA	Capuano et al. (2013)
*spvC*		570	F – ACTCCTTGCACAACCAAATGCGGA R – TGTCTTCTGCATTTCGCCACCATCA	
*pefA*	66.5	157	F – GCGCCGCTCAGCCGAACCAG R – GCAGCAGAAGCCCAGGAAACAGTG	Skyberg et al. (2006)
*sifA*		449	F- TTTGCCGAAGAACGCGCCCCCCACACG R- GTTGCCTTTTCTTGCGCTTTCCACCCATCT	
*gipA*	58	212	F – GCAAGCTGTACATGGCAAAG R – GGTATCGGTGACGAACAAAT	Capuano et al. (2013)
*sodC1*	50	467	F – TATTGTCGCTGGTAGCTG R – CAGGTTTATCCGAGTAAT	Capuano et al. (2013)
*gtgE*		1113	F – AGGAGGAGTGTAAAGGT R – GTAGAACTGGTTTATGAC	Ntivuguruzwa (2016)
*mig5*	58	248	F – AACCAACCAGACCAACCTTC R – GCAATACTGTTGCGCTTCTG	Capuano et al. (2013)
*rcK*		189	F – AACGGACGGAACACAGAGTC R – TGTCCTGACGAAAGTGCATC	Ntivuguruzwa (2016)
*sspH2*	58	203	F – GCACAACTGGCTGAAGATGA R – TTTCCCAGACGGAACATCTC	Capuano et al. (2013)
*srgA*		344	F – TGTTCCGGTCATAATGCAGA R – TTTTGAGGCCATCGAATACC	

Source: Please see the full reference list of the article Moatshe, N., Seakamela, E. & Mbatha, K.R., 2025, ‘Resistance, virulence and genetic diversity of *Salmonella* Typhimurium in South Africa (1999 -2021)’, *Onderstepoort Journal of Veterinary Research* 92(1), a2217. https://doi.org/10.4102/ojvr.v92i1.2217, for more information

bp, base pair.

### Detection of plasmids of *Salmonella* Typhimurium

Plasmid DNA was extracted using the ZymoPURE plasmid midiprep kit (Zymo Research, US) as per manufacturer’s instructions. Extracted plasmid DNA was run on a 0.7% agarose gel (Thermo Fisher Scientific, US) at 120 V for 1 h and visualised under UV light and photographed using an Omega Fluor gel documentation system (Omega Fluor, Aplegen). The Inqaba Biotechnologies (Pretoria, South Africa) extended 1 kb ladder and the *E. coli* 0157:H7 were used as the DNA marker and positive control, respectively.

### ERIC polymerase chain reaction

The ERIC PCR method was performed as described by Almeida et al. (2016). Primers (Inqaba Biotechnical Industries (Pty) Ltd., Johannesburg, South Africa): ERIC-R: ATG AAG CTC CTG GGG ATT CAC and ERIC-F: AAG TAA GTG ACT GGG GTG AGC G were used. In brief, the 25 µl PCR reaction mixture comprised 12.5 µL of Taq 2× Master Mix RED (Ampliquor, Denmark), 2 µL (5 pmoL/µL) of each primer (Inqaba Biotechnical Industries (Pty) Ltd., South Africa), 4.5 µL UltraPure DNase/RNase-Free Distilled Water (Thermo Fisher Scientific, US) and 4 µL DNA template. A thermocycler (9700 Applied Biosystems, US) was set as follows: initial denaturation at 94 ^o^C for 5 min, followed by 40 cycles of denaturation at 94 ^o^C for 60 s, annealing at 40 ^o^C for 90 s and extension at 72 ^o^C for 60 s with a single cycle of final extension at 72 ^o^C for 7 min. The PCR products were electrophoresed in 1.5% agarose gel containing 4 µL ethidium bromide at 120 V for 60 min and images were captured using Omega Fluor gel documentation systems (Omega Fluor, Aplegen). A 1 kb-plus DNA ladder (Biolabs, New England, UK) and *S.* Typhimurium (accession number: SRX10785603) were used as the DNA marker and a positive control, respectively.

### Data analysis

The data were subjected to a Chi-square test using the Frequency Procedure (PROC FREQ) of Statistical Analysis System (SAS) statistical software version 9.4 (Clark 2004). The following formula was used to calculate:


ARI=A/NY, [Eqn 1]

where A is the total number of resistance determinants recorded in the population, N is the number of isolates in the population and Y is the total number of antibiotics tested (Andriyanov et al. 2021). ERIC PCR dendrogram and analysis were analysed using BioNumerics software 6.6 (Applied Maths NV, Belgium) using the Dice coefficient and the unweighted pair group (UPGMA) with arithmetic averages using 1% tolerance and 0.5% optimisation settings to analyse electrophoretic patterns. Clustering was identified using a similarity cut-off of 70%. For better analysis, the years in this study were categorised into four periods spanning 5 years: 1999–2004; 2005–2010; 2011–2015; 2016–2021.

## Results

### Confirmation

A total of 180 isolates were initially selected for this study, of which only 92.0% (*n* = 140) proved to be viable on culture media. Of the 140 (92.1%) isolates, 129 were confirmed as *S*. Typhimurium using PCR, while 11 isolates were identified as other *Salmonella* species. Among the confirmed isolates, 72.9% (*n* = 94) were isolated from animal (bovine, caprine, equine, ovine, porcine, poultry) samples while 12.4% (*n* =16), 5.4% (*n* = 7) and 9.3% (*n* = 12) were from food, the environment and feed samples, respectively.

### Antimicrobial susceptibility test and antibiotic resistance index

A total of 129 isolates were tested against 13 antimicrobial agents and showed high resistance to ciprofloxacin (*n* = 112; 86.8%), ceftriaxone (*n* = 89; 69.0%), piperacillin (*n* = 84; 65.1%), amikacin (*n* = 79; 61.2%), cephalothin (*n* = 66; 51.2%), gentamycin (*n* = 60; 46.5%) and tetracycline (*n* = 40; 31.0%) (Table 5). The antibiotic resistance results showed statistical significance (*p* < 0.0001) with the exception of ceftriaxone (*p* = 0.0658). The Antibiotic Resistance Index (ARI) of all tested isolates was less than 0.2. This indicates a relatively low level of antibiotic resistance, suggesting that most of the *S.* Typhimurium isolates were exposed to limited selective pressure from antibiotics in their respective environments.

**TABLE 5:** Antimicrobial resistance patterns.

Antibiotic class	Name of antibiotics	Interpretation in %		*p*
		Sensitive	Resistant	
Penicillins or β-lactams	Ampicillin (AMP)	75.9	24.1	< 0.0001
	Piperacillin (TZP)	34.9	65.1	< 0.0001
	Amoxycillin (AMC)	83.7	16.3	< 0.0001
Cephalosporins	Cephalothin (KF)	48.8	51.2	< 0.0001
	Cefoxitin (FOX)	86.0	14.0	< 0.0001
	Ceftriaxone (CRO)	31.0	69.0	0.0658
Aminoglycoside	Gentamycin (GN)	53.5	46.5	< 0.0001
	Amikacin (AK)	38.8	61.2	0.0001
Tetracyclines	Tetracycline (TE)	69.0	31.0	< 0.0001
Fluoroquinolones	Ciprofloxacin (CIP)	13.2	86.8	< 0.0001
Monobactams	Aztreonam (ATM)	76.7	23.3	< 0.0001
Sulphonamides	Trimethoprim (STX)	87.6	12.4	< 0.0001
Phenicols	Chloramphenicol (CHL)	70.5	29.5	< 0.0001

### Antimicrobial resistance trends over the years

**FIGURE 1:**
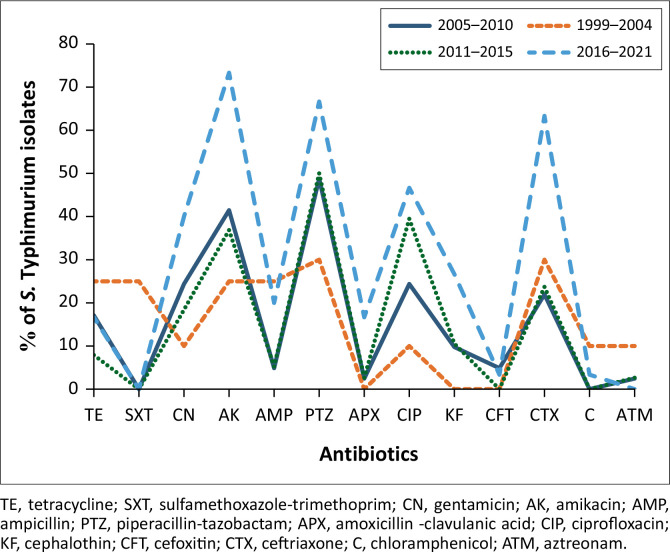
Antibiotic resistance trend of *Salmonella* Typhimurium from 1999 to 2021.

Antibiotic resistance by year showed that during the 1999–2004 period, piperacillin–tazobactam (30%) and ceftriaxone (30%) ranked higher followed by tetracycline, sulfamethoxazole-trimethoprim, amikacin and ampicillin with 25% each, while no resistance was observed in amoxicillin–clavulanic acid, cephalothin and cefoxitin. The year 2005–2010 was dominated by piperacillin–tazobactam (49%), amikacin (41%), gentamycin (24%), ciprofloxacin (24%) and ceftriaxone (22%) (Figure 1). During the third period (2011–2015), the highest resistance was observed in piperacillin–tazobactam (50%), ciprofloxacin (39%) and amikacin (37%) while the fourth period (2016–2021) was dominated by amikacin (73%), piperacillin–tazobactam (67%), ceftriaxone (63%) and ciprofloxacin (47%). Overall, sulfamethoxazole–trimethoprim, cefoxitin, chloramphenicol and aztreonam remained sensitive (≤ 5%) throughout the years while gentamycin and ciprofloxacin showed an increase from 10% to 47% in the period 1994–2001. Tetracycline remained constant throughout the years while resistance in amikacin, and piperacillin–tazobactam and ceftriaxone showed an increase from 25% to over 60% in 2021 (Figure 1).

### Virulence genes trends over the years

**FIGURE 2:**
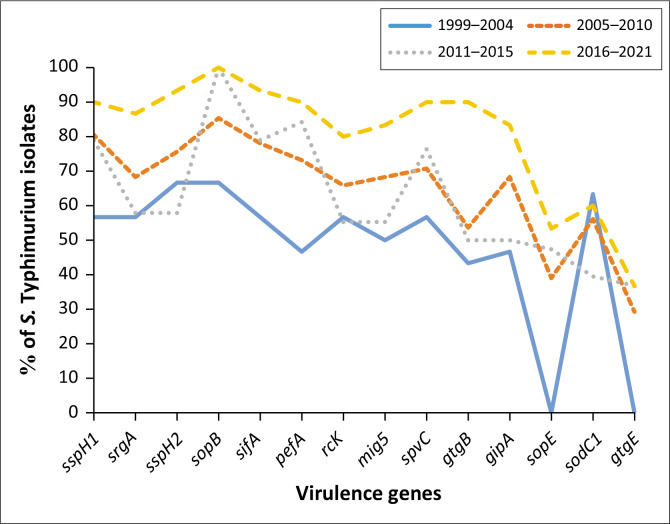
Virulence genes trend in *Salmonella* Typhimurium isolates from 1999 to 2021.

Figure 2 shows that during the period under investigation, all virulence genes, except *sopE* and *gtgE* were detected in over 40% – 67% in the first period (1999–2004) and increased steadily to over 80% in the fourth period (2016–2021).

### Antibiotic resistance patterns and multi-resistant isolates of *Salmonella* Typhimurium

Among the 129 isolates tested, 23 antimicrobial resistance patterns were observed. Resistance to three or more antimicrobials was seen across all species tested in this study. Only one isolate was resistant to 11 antimicrobials (TE, SXT, AK, AMC, CIP, KF, AMP, FOX, CHL, TZP and GN). At least 7.4% of porcine and 12.5% ovine isolates showed resistance to seven (AK–GN–TZP–CIP–FOX–KF–CRO) antibiotics. Resistance to the pattern AK–GN–FOX–KF–CIP–TZP (six antibiotics) was observed in 4.7% of poultry and 18.5% porcine isolates, while resistance to five antibiotics (GN–AK–TZP–CIP–KF) was observed in the following species: bovine (30.8%), poultry (13.9%), equine (11.1%) and other (25.0%). It was observed that 12.5%, 7.7%, 4.7% of isolates from ovine, bovine and poultry, respectively were resistant to four antimicrobials (TE–GN–AK–TZP). Resistance to three antibiotics was seen in the following species: bovine (11.5%), poultry (4.7%), porcine (14.8%), caprine (50.0%) and ovine (12.5%). Resistance to two antibiotics yielded three patterns: pattern 1 (TZP-CIP) – porcine (3.7%), caprine (25.0%), equine (11.1%), ovine (12.5%) and other (16.7%). Pattern 2 (AK-TZP) – porcine (14.8%), equine (11.1%) and ovine (12.5%). Pattern 3 (CIP-CRO) – bovine (11.5%), poultry (20.9%), porcine (14.8%) and equine (22.2%).

### Presence of antimicrobial resistant genes in *Salmonella* Typhimurium isolates

A total of 129 isolates were screened for the presence of 18 resistance genes. The *bla*_PSE_ gene was detected in 32.6% (*n* = 42) while *bla*_CMY-2_, *bla*_TEM_ and *bla*_SHV_ genes were present in 21.7% (*n* = 28), 18.6% (*n* = 24) and 17.5% (*n* = 23) of the isolates, respectively. Furthermore, isolates harboured 18.6 % (*n* = 24), 7.8% (*n* = 10) and 6.2% (*n* = 8) of *sul1, sul2* and *sul3* genes, respectively. The *qnrA* gene was detected in 20.2% (*n* = 26), followed by *qnrB* (21.7%; n = 28) and *qnrS* (10.9%; *n* = 14) while *tetA* and *tetB* were detected in 24.0% (*n* = 31) and 22.5% (*n* = 29) of the isolates, respectively. Among the trimethoprim encoding genes, resistance was detected in *dfrXI* (18.6%; *n* = 24) and *dfrXII* (15.5 %; *n* = 20), while no resistance was detected for *dfrxIII* (0.0%).

### Presence of integrons in *Salmonella* Typhimurium isolates

In this study, isolates were screened for class 1 (*int1*), 2 (*int2*) and 3 (*int3*) integrons with *int1* gene detected in 47.3% (*n* = 61) of the isolates, followed by *int2* (36.4%; *n* = 47) and *int3* (24.8%; *n* = 32) (class 3) encoding for the *int3* gene. Class 2 (*p* = 0.0021) and class 3 (*p*-value = 0.0001) results were considered statistically significant.

### Presence of virulence genes among *Salmonella* Typhimurium

Among the 129 *S*. Typhimurium isolates investigated in this study, *InvA* (100%; *n* =129) was predominant, followed by *sopB* gene (95.3%; *n* = 123) *sspH1* (82.9%, *n* =107), *sifA* (82.9%; *n* =107), *pefA* (79.8%; *n* = 103), *spvC* (79.1%; *n* = 102), *sspH2* (77.5%; *n* =100) and *srgA* (71.3%; *n* =92). Furthermore, the *mig5* and *rcK* genes were both detected in 69.0% while *gipA, gtgB, sopE, sodC1* and *gtgE* were detected in 66.7%, 62.8%, 60.5%, 58.1% and 19.4%, respectively.

### Presence of plasmids in *Salmonella* Typhimurium isolates

Conducting plasmid extraction from 129 *S*. Typhimurium isolates, revealed the presence of a diverse range of plasmid sizes from 2 kb to 90 kb. Remarkably, among these isolates, a significant majority, constituting 71.3% (*n* = 92), were found to possess the substantial 90 kb plasmid. In contrast, only a small fraction, 2.3% (*n* = 3) of the isolates, carried the diminutive 2 kb plasmid, while a noteworthy 31.0% (*n* = 40) of isolates contained the intermediate-sized 8 kb plasmid.

### ERIC polymerase chain reaction

**FIGURE 3:**
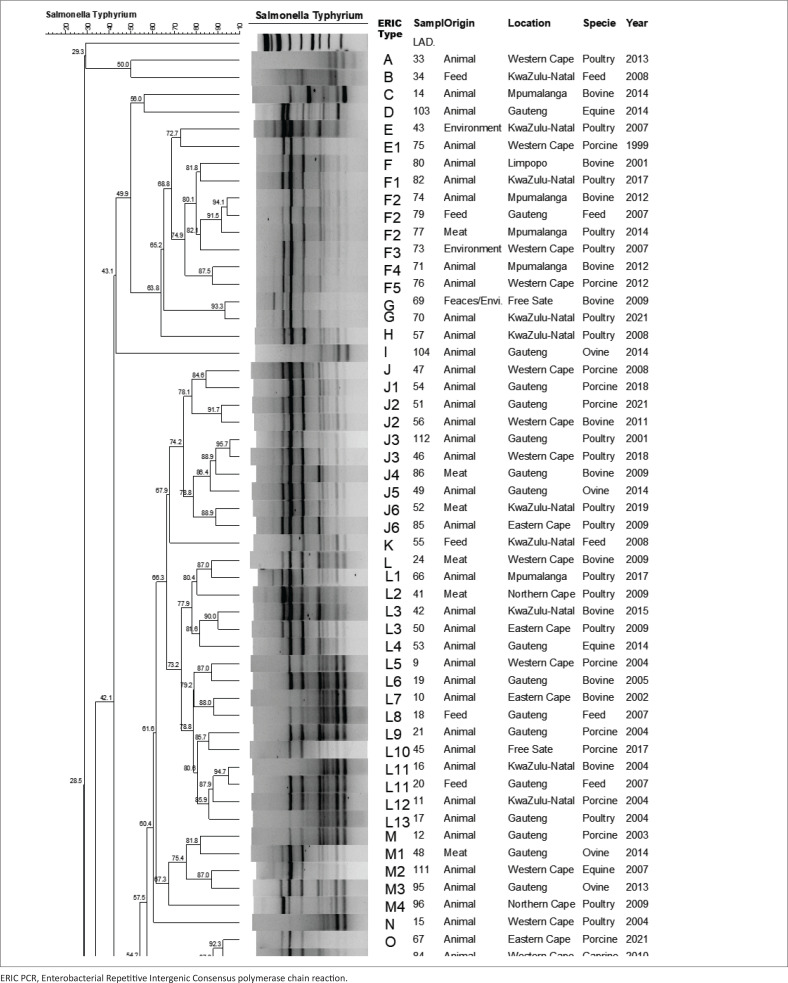

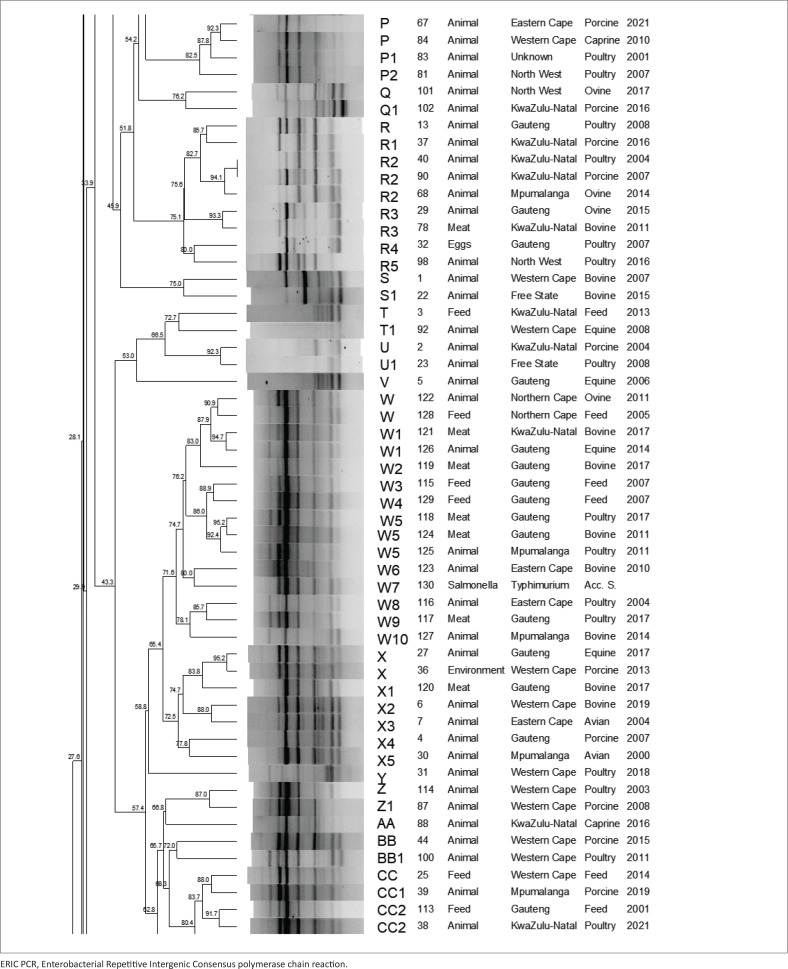

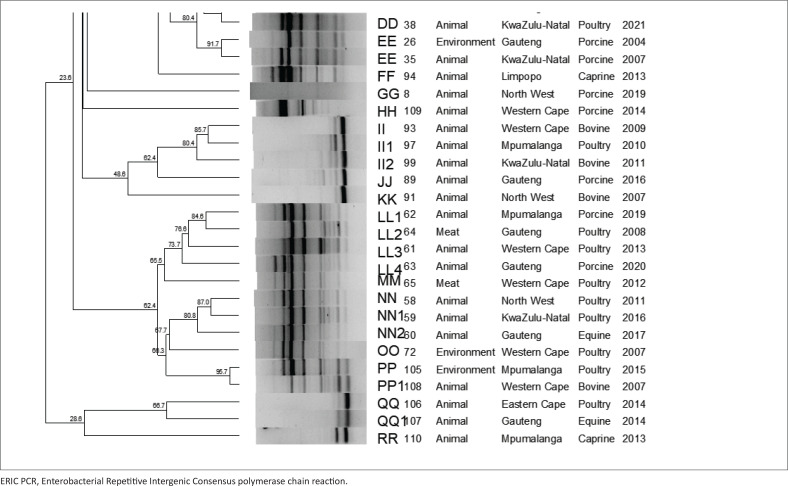
A dendrogram illustrating the genetic similarity, source, sample type and year of *S.* Typhimurium isolates. The ERIC PCR cluster grouping is based on a cluster index of 70% and similarity index of 90%. A dendrogram illustrating the genetic similarity, source, sample type and year of *S.* Typhimurium isolates. The ERIC PCR cluster grouping is based on a cluster index of 70% and similarity index of 90%. A dendrogram illustrating the genetic similarity, source, sample type and year of *S.* Typhimurium isolates. The ERIC PCR cluster grouping is based on a cluster index of 70% and similarity index of 90%.

The DNA fingerprinting of 129 isolates was done using ERIC PCR to determine the relationship of isolates (Figure 3). Furthermore, isolates were grouped into clusters based on a cluster index of 70% and clonal relation was determined at a similarity index of 90%. The isolates were categorised into 44 different ERIC types (A – RR), with six major clusters being identified: F (*n* = 8), J (*n* = 10), L (*n* = 16), R (*n* = 9), W (*n* =1 4) and X (*n* = 7). Clonal relatedness (three or more isolates) was observed among the isolates in clusters F, J, L, R, W and CC. It was also noted that 80% (*n* = 8) of isolates from cluster J were from animal sources (porcine [*n* = 3], bovine [*n* = 1], ovine [*n* = 1] and poultry [*n* = 3]) and meat (*n* = 2). In cluster L, 75% (*n* = 12), 12.5% (*n* = 2) and 12.5% (*n* = 2) isolates were from animals [porcine (*n* = 4), bovine (*n* = 4), equine (*n* =1) and poultry (*n* = 3)], meat and feed, respectively.

Cluster W had 42.9% (*n* = 6) isolates from animals (equine [*n* =1], bovine [*n* = 2], ovine [*n* = 1] and poultry [*n* = 2]), 35.7% (*n* = 5) from meat and 21.4% (*n* = 3) from feed. Among these isolates, 50% (*n* = 7) were isolated from Gauteng province; moreover, 50% (*n* = 7) isolates were clonally related. Cluster F consisted of 62.5% isolates originating (*n* = 5) from animals (porcine [*n* = 1], bovine [*n* = 3] and poultry [*n* = 1], 12.5% [*n* = 1] from environment, 12.5% [*n* = 1] from feed and 12.5% [*n* = 1]) from meat and X consisting of 71.4% isolates (*n* = 5) from animals (porcine [*n* = 1], bovine [*n* = 1], equine [*n* = 1] and poultry [*n* = 2]), 14.3% (*n* = 1) meat and 14.3% (*n* = 1) environment. Cluster R consisted of 77.8% (*n* = 7) of isolates taken from animals (poultry [*n* = 3], porcine [*n* = 2] and ovine [*n* = 2]). Furthermore, in cluster R, two isolates indicated 100% similarity and were both from KwaZulu-Natal (Figure 3).

## Discussion

In the current study, 12% – 87% of isolates showed resistance to 13 antibiotics, with the highest proportion towards ciprofloxacin (86.8%), ceftriaxone (69.0%), piperacillin (65.1%), amikacin (61.2%), cephalothin (51.2%), gentamycin (46.5%) and tetracycline (31.0%). Salmonellosis caused by *S.* Typhimurium is self-limiting in healthy individuals. However, systemic infections in young children, the elderly and immunocompromised individuals require antimicrobial treatment (Nazir et al. 2025). The recommended regimen for *Salmonella* includes third-generation cephalosporins, quinolones and macrolides (Collignon et al. 2016).

Resistance towards ciprofloxacin in this study was observed in 86.8% of the isolates. Ciprofloxacin is an antibiotic of choice for the treatment of invasive *Salmonella* infections in adults (Parry & Threlfall 2008). The result of the current study exceeds the 64%, 19.5% and 0% reported in China, Iran and Turkey, respectively (Guo et al. 2023; Moghadam et al. 2023; Şik & Akan 2024). However, the result of our study was lower than the result (100%) reported by Siddiky et al. (2024) in Bangladesh.

Sixty-nine per cent and 51.6% of the isolates showed resistance towards ceftriaxone and cephalothin, respectively. Cephalothin, a first-generation cephalosporin is known to treat serious infections caused by both Gram positive and negative bacteria including *Salmonella* while ceftriaxone is an empirical choice for the treatment of *Salmonella* infections in children (Shi et al. 2021). Furthermore, ceftriaxone is a preferred antibiotic for invasive infections when bacteria are resistant to ciprofloxacin (Arizpe et al. 2016). The resistance towards cephalothin in our study was found to be higher than 0% reported in Malaysia by Adzitey, Rusul and Huda (2012), but lower than 75.7% reported by Elshebrawy et al. (2022) from Egypt, while the high and low resistance towards ceftriaxone in comparison to our study was reported from Nigeria (100.0%) and Bangladesh (13.8%), respectively (Igbinosa et al. 2023; Rahman et al. 2024).

Resistance to piperacillin was recorded in 50.4% of the isolates. Consequently, the presence of piperacillin-resistant *S*. Typhimurium in non-human sources raises the possibility of a hypothetical transmission from human strains to non-human origins. The current results were found to be lower than the 100% rate reported in *S*. Typhimurium isolates by Igbinosa et al. (2023). The disparity in study results can be attributed to variations in antibiotic usage patterns influenced by geographical location. However, Moghadam et al. (2023) reported results (58.3%) similar to this study in Iran.

In terms of aminoglycosides, resistance was observed in amikacin (61.2%) and gentamycin (46.5%). Amikacin and gentamycin are critically important antibiotics used to treat enterococcal endocarditis and MDR tuberculosis (WHO 2007). Resistance to these antibiotics may be fuelled by their use in treating animal infections. The resistance in our study towards amikacin and gentamycin was lower than those reported in a study from Italy which reported 100.0% resistance towards each antibiotic while studies in Egypt and Bangladesh reported 10.8% and 13.3%, respectively (Elshebrawy et al. 2022; Siddiky et al. 2024).

In this study, 31.0% of isolates were resistant to tetracycline. Tetracycline is a broad-spectrum antibacterial agent used in both human and veterinary medicine to treat and prevent *Salmonella* infections (Lugo-Melchor et al. 2010). The use of tetracycline to promote growth in animals resulted in an increase in resistant isolates. However, high levels of resistance to tetracycline were reported in studies from Iran: 72.2% by Moghadam et al. (2023), Bangladesh: 86.6% by Siddiky et al. (2021) and Malaysia: 78.4% by Adzitey et al. (2012). The results of the current study are in correlation with those reported in Egypt: 32.4% (Elshebrawy et al. 2022). The extensive use of tetracycline worldwide has resulted in the development of resistance among *Salmonella* spp. (Pavelquesi et al. 2021).

The high resistance towards critically and highly important antimicrobials in this study is of great concern as it may impair treatment efficacy (Ahmed et al. 2024). Furthermore, antimicrobial resistance in *Salmonella* is associated with horizontal gene transfer which may influence the pathogenic characteristics of *Salmonella* strains in the future (Collignon et al. 2016). It is worth noting that the use of antibiotics as growth promoters has been banned in the European Union in 2006 (Henton et al. 2011). However, the use of antibiotics for animal production in African countries including South Africa continues to date. Although there is a move on firm awareness on the usage of antibiotics in animal production in South Africa, stringent measures need to be taken to remedy this situation.

During the period under investigation, the antibiotic and virulence trends of isolates in our study showed low proportion in the early 2000s with steady increase in both the resistance and virulence which was as expected. Our results were consistent with the previous studies that reported the resistance of *Salmonella* species to commonly used antibiotics including tetracyclines even beyond 1999; hence most of the isolates were already showing resistance towards most of the antibiotics tested (Ali et al. 2025; Hur, Jawale & Lee 2012; Nonga et al. 2010). Furthermore, our study supports the understanding that antimicrobial resistance is a complex process that is fuelled by many factors including the misuse and overuse of antibiotics in both human and veterinary medicine. Therefore, prioritising antimicrobial stewardship is essential.

One isolate from poultry was resistant to 11 antibiotics (TE–SXT–AK–AMC–CIP–KF–AMP–FOX–CHL–TZP–GN). Siddiky et al. (2021) found one *S.* Typhimurium isolate to be resistant to 12 antibiotics which exceeded our results. However, a study in Italy found one isolate to be resistant to 10 antibiotics (Lauteri et al., 2022). The notable rise in resistance to critical antibiotics such as ciprofloxacin, third-generation cephalosporins and aminoglycosides emphasises the urgent need for prudent antibiotic use and effective surveillance to address antibiotic resistance effectively (O’neil 2016). In addition, these results highlight the significance of ongoing monitoring of antibiotic susceptibility to guide optimal treatment plans and public health interventions (WHO 2017). Isolates in this study were MDR, with an increase over the years.

Resistance to β-lactams in *S. enterica* is mainly because of the production of acquired β-lactamases. In the current study, *bla*_PSE_ and *bla*_TEM_, which are frequently associated with the ampicillin and amoxicillin/clavulanate resistance were detected in 32.6% and 18.6% while *bla*_CMY-2,_ and *bla*_SHV_ genes which code for the resistance of third-generation cephalosporins and penicillins were present in 21.7% and 17.5%, respectively (De Toro et al. 2011; Zhao et al. 2009). Zhao et al. (2017) and Oh et al. (2016) reported 12.5% and 1.1% *bla*_PSE_ detection, respectively, which were lower than our results. The *bla*_TEM_ results in our study were lower than those reported by studies in Pakistan (100%) and Bangladesh (64.8%) (Fatima et al. 2023; Rahman et al. 2024). In this study, the *bla*_CMY-2_ detection was higher than those reported by Thong and Modarressi (2011) in Malaysia while the *bla*_SHV_ was reported in 12.5% of the isolates by Rahman et al. (2024). The presence of *bla* genes suggest that the *S*. Typhimurium isolates encoding these genes can effectively resist β-lactams. Furthermore, these genes can be transferred to other isolates.

In the current study, *sul1, sul2* and *sul3* genes, which are responsible for conferring resistance to sulphonamides, were detected in 18.6 %, 7.8% and 6.2% of the isolates, respectively. These results were consistent with the phenotypic resistance profiles. In a study conducted in Iran, *sul1, sul2* and *sul3* were detected in 84%, 50% and 17%, respectively, which were higher than the results of our study (Moghadam et al. 2023).

Tetracycline-encoding *tetA* and *tetB* genes were detected in 24.0% and 22.5% of *Salmonella* isolates, respectively, aligning with the observed phenotypic resistance patterns. These genes are responsible for encoding efflux pumps that actively expel tetracycline antibiotics from bacterial cells, thereby lowering the intracellular concentration of the antibiotic and diminishing its efficacy (Møller et al., 2016; Pavelquesi et al. 2021). It was observed that the occurrence of *tetA* and *tetB* genes in *Salmonella* is a consequence of intricate interactions involving various factors, such as antibiotic utilisation, bacterial genetics and environmental conditions (Mthembu et al. 2021). For instance, Moghadam et al. (2023) reported notably varying detection rates of 72% for *tetA* and 23% for *tetB* genes in tetracycline-resistant *Salmonella* Typhimurium isolates in Iran. Lauteri et al. (2022) assessed antimicrobial resistance in *Salmonella* Typhimurium strains isolated from Italian swine food chain, with *tetA* and *tetB*, genes identified in 26.3% and 73.7% of these isolates, respectively.

The emergence of plasmid-mediated fluoroquinolone resistance in *Salmonella* is driven by the presence of *qnrA, qnrB* and *qnrS* genes. In the current study, it was observed that 21.7%, 20.2% and 10.8% of *S*. Typhimurium isolates carried the *qnrB, qnrA* and *qnrS* genes, respectively. These findings contrast with those reported by Zhao et al. (2017) who detected *qnrA* and *qnrB* in 81.3% of the isolates for each. However, Zhao et al. (2017) and Pribul et al. (2017) observed a *qnrS* detection of 3.1% and 3.6%, respectively which was lower than our results. Importantly, the occurrence of *qnrA, qnrB* or *qnrS* genes in *Salmonella* strains can vary significantly depending on geographic location and *Salmonella* serotype. Despite the presence of these genes associated with quinolone and fluoroquinolone resistance in this study, the phenotypic results indicated a high level of resistance to ciprofloxacin. This discrepancy might be attributed to the absence of an efficient promoter region, or the possibility of these genes conferring greater resistance or susceptibility to ciprofloxacin. However, the presence of *qnrA, qnrB* and *qnrS* genes in our isolates is of concern, especially as quinolones are approved for therapeutic and preventive use in animals in South Africa (Eagar, Swan & Van Vuuren 2012). Therefore, their misuse could contribute to the emergence of antibiotic-resistant bacteria in humans (Henton et al. 2011).

In this study, the presence of *sifA* (82.9%) and *sopB* (95.3%) were detected within *S*. Typhimurium isolates which agrees with the findings of other studies (Hughes et al. 2008; Skyberg et al. 2006). These genes play pivotal roles in the invasion of macrophages, with *sifA* being particularly responsible for the pathogen’s survival, contributing to *Salmonella* infection (Ibarra & Steele-Mortimer 2009).

Plasmid encoding virulence genes (*mig5, rcK, srgA, spvC* and *pefA*) were detected in a range of 69.0% – 79.8% of the isolated strain. These findings were higher than results reported by Proroga et al. (2019) and Capuano et al. (2013) in Italy. The *spv* gene encodes the main components for *S.* Typhimurium plasmid-mediated virulence and is carried into host cells T3SS2 (Browne et al. 2008; Ibarra & Steele-Mortimer 2009). These genes (*rck, SrgA, SpvC, PefA*) are involved in infection stages such as adhesion, invasion, adaptation, intracellular survival within host macrophages and also the evasion of host immune responses (Ahmer, Tran & Heffron 1999; Kendall & Sperandio 2014; Koczerka et al. 2021; Long et al. 2022).

Bacteriophages encoding virulence genes *gtgB* and *sodC1* were detected in 71.4% and 68.6% of the isolated strains, respectively, while 19.3% of the isolates carried *gtgE*. These findings diverge slightly from those reported by Capuano et al. (2013), where *gtgE* was absent in all the isolates. These genes (*gtgE, gtgB* and *sodC1*) facilitate *Salmonella’s* survival within a host and enhance its pathogenicity, as described by Foley et al. (2013), making *Salmonella* infections challenging to effectively treat.

In this study, the presence of the Gifsy-1 encoded gene *gipA*, which aids *Salmonella* in invading host cells, was detected in 66.7% of the isolates (Nguyen Thi et al. 2020). These findings align with the observations made by Capuano et al. (2013) and Proroga et al. (2019) in Italy. However, current results are lower than the 1.43% reported by Sharma et al. (2019) in chickens. In addition, this study revealed the presence of the *sspH1* gene in 82.9% of the *S*. Typhimurium isolates, which is higher compared to the findings reported by Capuano et al. (2013) but consistent with the results reported by Long et al. (2022). The elevated prevalence of these virulence genes in our study underscores the potential of the isolated *S.* Typhimurium strains to cause infections.

The 90 kb plasmid known as the virulence plasmid that primarily functions to boost *Salmonella’*s growth during the systemic phase of the illness was recorded in 71.3% of isolates in this study (Gulig & Doyle 1993). This plasmid is capable of mobilisation but lacks conjugative abilities (Ahmer et al. 1999). These results were lower than the 44.0% (*n* = 16/46) reported by Sameshima et al. (2000) in Japan and 48.5% reported by Benacer et al. (2010) in Malaysia. However, no isolates were reported to carry this plasmid by Guerra et al. (2002) in Spain. In a study by Khasa, Singh and Sidhu (2018) in India, 100% of *S*. Typhimurium isolates carried the 90 kb plasmid.

The ERIC PCR fingerprinting has been shown to provide increased discrimination and is fast, simple and cheap (Kumar, Surendran & Thampuran 2008). Using ERIC PCR, the *S*. Typhimurium isolates were differentiated and grouped into 44 clusters (A-RR). In this study, Cluster F consisted of isolates originating from feed, meat, environment and animals and cluster X consisted of isolates originating from animals, environment and meat. This could indicate a possible cross-contamination among the different sources of isolation although these isolates were collected from different provinces. All isolates in cluster J originated from animals and were widely distributed between 2001 and 2021. Understanding the source of *Salmonella* in the food chain is crucial for minimising occurrences in humans (Magwedere et al. 2015). The clonal relatedness among isolates (50%) in cluster W suggests that isolates from different provinces in South Africa are related. Cluster L consisted of isolates from animals and feed from Gauteng province. These findings suggest that there might be a cross-contamination between animals and feed in Gauteng province. In cluster R, a pair of clones were reported from KwaZulu-Natal in different species. Findings in this study suggest that *S*. Typhimurium is widely distributed and this is in agreement with studies by Gelaw, Nthaba and Matle (2018) and Magwedere et al. (2015).

These findings highlight the urgent need for robust antimicrobial stewardship programmes and stringent regulatory measures to control antibiotic usage in both health care and agricultural practices. In addition, continuous surveillance and monitoring of antimicrobial resistance patterns are critical to inform policy decisions and implement effective interventions aimed at curbing the spread of resistant *S.* Typhimurium strains. Addressing this issue is paramount to safeguarding public health and ensuring the continued efficacy of vital antimicrobial therapies.

## Conclusion

*Salmonella* Typhimurium remains a significant global foodborne pathogen with profound implications for both public and animal health. This study has demonstrated that *S*. Typhimurium isolates in South Africa exhibit resistance to critically and highly important antibiotics including ciprofloxacin, which is a cornerstone of treatment for invasive salmonellosis. In addition, most isolates were found to harbour virulence factors associated with adhesion, invasion and intracellular survival. The combination of antimicrobial resistance (AMR) and pathogenicity factors highlights the dual threat posed by these isolates, making them a critical public health risk. Therefore, the authors recommend the implementation of stringent antimicrobial stewardship policies in both human and veterinary medicine as well as robust surveillance programmes to monitor the epidemiology of *S*. Typhimurium and other foodborne pathogens.
